# Crystal structure of 2-azido-1,3-bis­(2,6-diiso­propyl­phen­yl)-1,3,2-di­aza­phospho­lidine

**DOI:** 10.1107/S2056989017007642

**Published:** 2017-05-31

**Authors:** Alex J. Veinot, Amber D. Blair, Jason D. Masuda

**Affiliations:** a Department of Chemistry, Saint Mary’s University, 923 Robie St., Halifax, Nova Scotia, B3H 3C3, Canada

**Keywords:** crystal structure, N-heterocyclic phosphine, NHP, 2-azido-1,3,2-di­aza­phospho­lidine

## Abstract

The title compound, C_26_H_38_N_5_P, was synthesized by reacting 2-chloro-1,3-bis­(2,6-diiso­propyl­phen­yl)-1,3,2-di­aza­phospho­lidine with sodium azide and a catalytic amount of lithium chloride in tetra­hydro­furan. The title compound is the first structurally characterized 2-azido-1,3,2-di­aza­phospho­lidine and exhibits a P atom in a trigonal pyramidal geometry. The azide P—N bond length of 1.8547 (16) Å is significantly longer than the P—N separations for the chelating di­amine [P—N = 1.6680 (15) and 1.6684 (14) Å]. The sterically hindered 2,6-diiso­propyl­phenyl groups twist away from the central heterocycle, with dihedral angles between the central heteocyclic ring and benzene rings of 76.17 (10) and 79.74 (9)°. In the crystal, a weak C—H⋯N link to the terminal N atom of the azide group leads to [100] chains.

## Abstract 2

The synthesis, spectroscopic and crystal structure of 2-azido-1,3-bis­(2,6-diiso­propyl­phen­yl)-1,3,2-di­aza­phospho­lidine is reported.

## Chemical context   

Phosphine azides possess at least one azide group attached to phospho­rus and display a broad range of reactivity that is directly dependent on the other substituents attached to the P atom. One of the most inter­esting properties of these mol­ecules is that both free and coordinated alkyl and aryl derivatives are much more reactive than their corresponding amino derivatives, as demonstrated by their lower thermal and photochemical stability. For example, the phosphinoazide complex Ph_2_P(N_3_)–Cr(CO)_5_ readily undergoes photolysis under UV light to produce the phosphino–iso­cyanate complex Ph_2_P(NCO)–Cr(CO)_5_ (Ocando *et al.*, 1985 ▸), while the related bis­(diiso­propyl­amino) complex (iPr_2_N)_2_P(N_3_)–Cr(CO)_5_ does not (Cowley *et al.*, 1995 ▸). The crystal structure of the title compound is the first reported example of a structurally characterized 2-azido-1,3,2-di­aza­phospho­lidine; however, a few closely related compounds are known, such as those derived from 1,3,2-di­aza­phospho­lenes.
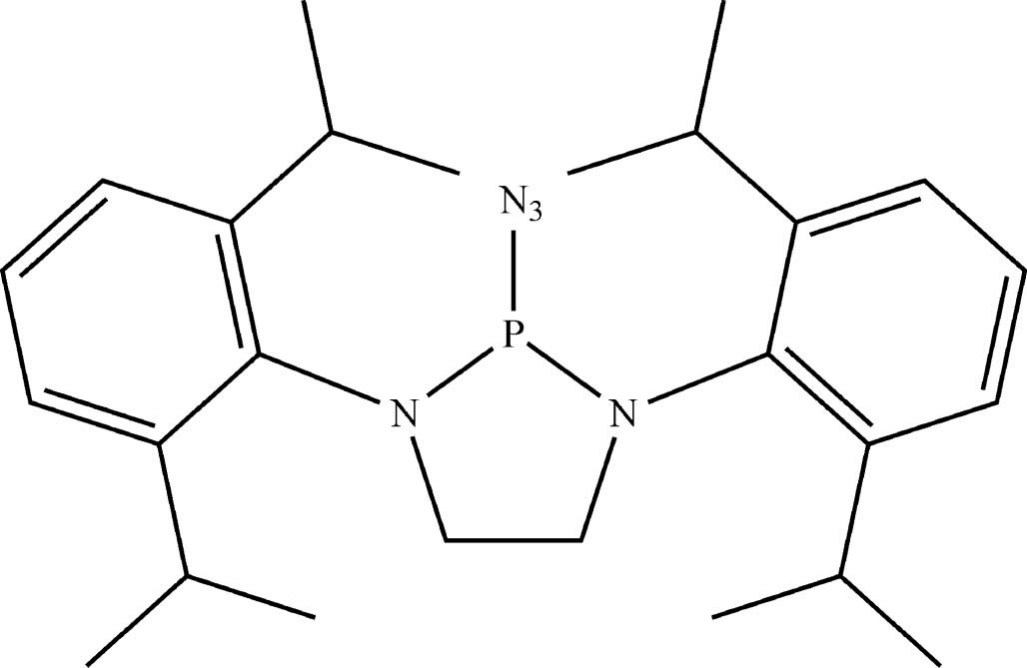


## Structural commentary   

**Figure 1:**
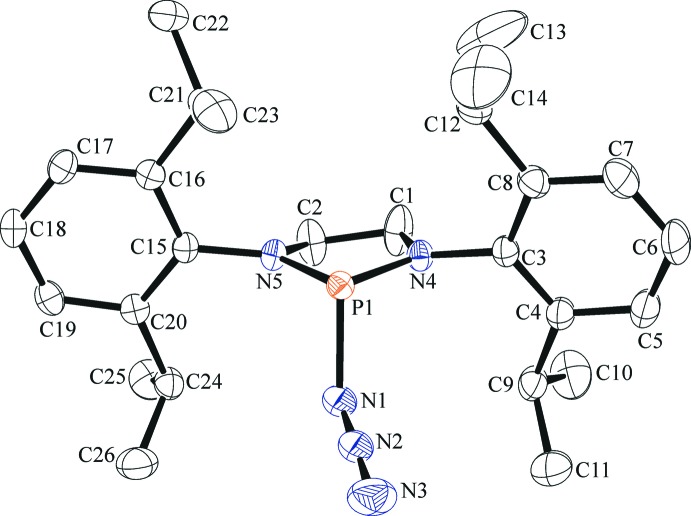
The mol­ecular structure of the title compound, showing 50% displacement ellipsoids. H atoms have been omitted for clarity.

The mol­ecular structure of the title compound is shown in Fig. 1 ▸. It crystallizes in the monoclinic space group *P*2_1_/*n* with one mol­ecule in the asymmetric unit. The bond lengths between the P atom and its flanking N atoms are similar [P1—N4 = 1.6680 (15) Å, P1—N5 = 1.6684 (14) Å and N4—P1—N5 = 91.14 (7)°], while the phospho­rus centre adopts a trigonal pyramidal geometry, with the sum of the angles at phospho­rus equal to 294.14 (7)°. The azide group is quasilinear [N3—N2—N1 = 176.6 (2)°], with similar N—N bond lengths [N1—N2 = 1.168 (2) Å and N2—N3 = 1.155 (2) Å]. The phospho­rus–azide bond length (P1—N1) is significantly longer [1.8547 (16) Å] than found for atoms N4 and N5. The average sum of the bond angles at the N4 and N5 positions is 359.87 (12)°, very close to an ideal trigonal planar geometry. This is a strong indication that the nominal lone pairs of atoms N4 and N5 participate in N—P⋯π inter­actions and, when coupled with the significantly longer P1—N1 bond length, suggests a partial ionic character similar to earlier reports in acyclic structures (Cowley *et al.*, 1995 ▸). The overall conformation of the C1/C2/N4/N5/P1 ring is well described as an envelope, with atom N5 deviating from the other atoms (r.m.s. deviation = 0.030 Å) by −0.274 (2) Å. The steric demands of the bulky 2,6-diiso­propyl­phenyl groups cause the aromatic rings to twist away from the central five-membered ring, with torsion angles of 103.69 (18) and 101.83 (17)° for P1—N1—C3—C4 and P1—N2—C15—C20, respectively. The isopropyl groups are oriented away from the central five-membered ring, but the ‘congested’ nature of the mol­ecule results in intra­molecular short contacts between all four of the methine H atoms (H9, H12, H21 and H24) and atoms N4 and N5 (Table 1 ▸).

## Supra­molecular features   

**Figure 2:**
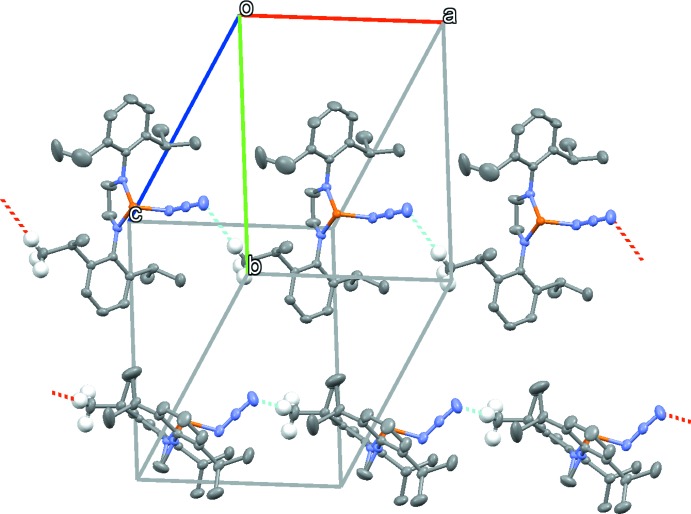
The packing of the title compound, showing inter­molecular C—H⋯N inter­actions as dashed lines, which result in [100] chains.

The only significant directional inter­action in the crystal of the title compound is a long [2.69 (3) Å] C—H⋯N hydrogen bond to the terminal N atom of the azide group, which results in [100] chains in the crystal (Fig. 2 ▸).

## Database survey   

A search of the Cambridge Structural Database (Groom *et al.*, 2016 ▸) indicated that no other 2-azido-1,3,2-(di­aryl­amino)­phospho­lidine derivatives have been structurally characterized. Some structurally similar compounds were identified, however, namely 2-azido-1,3-bis­(2,6-diiso­propyl­lphen­yl)-1,3,2-di­aza­phospho­lene (CSD refcode CILBAC; Gediga *et al.*, 2014 ▸) and its corresponding 2,6-di­methyl­phenyl derivative (GOFHAL; Burck *et al.*, 2008 ▸). Acyclic derivatives featuring bis­(diiso­propyl­amino) (PIJZAJ; Englert *et al.*, 1993 ▸) and bis­(dicylo­hexyl­amino) (ZABCEK; Cowley *et al.*, 1995 ▸) ligands are known, and also 1-azido-*N*,*N*′-bis(2,4,6-tri-*tert*-butyl­phenyl)phosphinedi­amine (YABVUV; Nieger *et al.*, 2016 ▸).

## Synthesis and crystallization   

The synthesis of the title compound was achieved using a similar method as reported in the literature for 2-azido-1,3-bis­(2,6-diiso­propyl­lphen­yl)-1,3,2-di­aza­phospho­lene (Gediga *et al.*, 2014 ▸). In a 20 ml scintillation vial, 0.102 g (0.229 mmol, 1 eq.) of colourless 2-chloro-1,3-bis­(2,6-diiso­propyl­phen­yl)-1,3,2-di­aza­phospho­lidine were dissolved in 1 ml of THF producing a colourless solution. To this solution, 0.016 g (0.246 mmol, 1.1 eq.) of colourless sodium azide and a spatula tip (<1 mg) of lithium chloride were added to solution immediately producing a colourless mixture. The reaction mixture was left to stir for 1 d and monitored using ^31^P{^1^H} NMR spectroscopy, and once the starting material was completely consumed the reaction mixture was dried *in vacuo*. Extraction of the colourless residue with cold pentane, followed by filtration through Celite produced a colourless solution, which afforded 0.060 g (60%) of the title compound as a colourless powder after removal of the solvent. Crystals of the product were obtained by concentrating the filtrate and storing in a 238 K freezer overnight. ^1^H NMR (CDCl_3_): δ 7.31 (*t*, ^3^ *J*_HH_ = 7.6 Hz, 2H, *p*-Dipp), 7.24–7.17 (*m*, 4H, *m*-Dipp), 3.88–3.82 (pseudo-*q*, 2H, NHC-CH_2_), 3.74 (*sept*, ^3^ *J*_HH_ = 6.8 Hz, 2H, iPr-CH), 3.48–3.39 (*m*, 4H, iPr-CH, NHC-CH_2_), 1.33–1.25 (*m*, ^3^ *J*_HH_ = 6.8 Hz, 24H, iPr-CH_3_). ^13^C{^1^H} NMR (CDCl_3_): δ 150.3, 148.4, 136.2, 128.1, 124.7, 124.2, 54.4, 29.0, 25.3, 24.9, 24.5. ^31^P{^1^H} NMR (CDCl_3_): δ 129.8. IR (KBr pellet): ν 3062 (*w*), 2963 (*s*), 2926 (*m*), 2867 (*m*), 2500 (*w*), 2125 (*m*), 2085 (s, N=N=N), 1678 (*w*), 1584 (*w*), 1462 (*s*), 1445 (*s*), 1383 (*m*), 1363 (*m*), 1324 (*m*), 1323 (*m*), 1257 (*s*), 1211 (*w*), 1185 (*w*), 1106 (*m*), 1075 (*s*), 1056 (*w*), 1043 (*w*), 980 (*w*), 946 (*w*), 935 (*w*), 852 (*w*), 806 (*s*), 761 (*s*), 730 (*w*), 688 (*w*), 651 (*w*), 602 (*w*), 583 (*w*), 550 (*w*), 542 (*w*), 470 (*s*), 437 cm^−1^ (*w*). M.p. (K): 415.4–417.6 (decomposes, gas was released).

## Refinement   

Crystal data, data collection and structure refinement details are summarized in Table 2 ▸. H atoms were included in geometrically idealized positions and refined using a riding model. Dihedral angles for the methyl H atoms were allowed to refine freely. The atomic displacement parameters of atoms N1 and N2 were constrained to be approximately equal using an EADP command.

## Supplementary Material

Crystal structure: contains datablock(s) I. DOI: 10.1107/S2056989017007642/hb7680sup1.cif

Structure factors: contains datablock(s) I. DOI: 10.1107/S2056989017007642/hb7680Isup2.hkl

Supporting information file. DOI: 10.1107/S2056989017007642/hb7680Isup3.cml

CCDC reference: 1551849

Additional supporting information:  crystallographic information; 3D view; checkCIF report

## Figures and Tables

**Table 1:** Hydrogen-bond geometry (Å, °)

*D*—H⋯*A*	*D*—H	H⋯*A*	*D*⋯*A*	*D*—H⋯*A*
C9—H9⋯N4	1.00	2.43	2.926 (2)	110
C12—H12⋯N4	1.00	2.44	2.913 (2)	109
C21—H21⋯N5	1.00	2.49	2.932 (2)	106
C24—H24⋯N1	1.00	2.66	3.443 (3)	136
C24—H24⋯N5	1.00	2.46	2.955 (2)	110
C22—H22*C*⋯N3^i^	0.98	2.69	3.669 (3)	174

Symmetry code: (i) 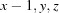.

**Table 2:** Experimental details

Crystal data	
Chemical formula	C_26_H_38_N_5_P
*M*_r_	451.58
Crystal system, space group	Monoclinic, *P*2_1_/*n*
Temperature (K)	150
*a*, *b*, *c* (Å)	10.0148 (12), 17.343 (2), 15.6270 (19)
β (°)	105.948 (2)
*V* (Å^3^)	2609.7 (5)
*Z*	4
Radiation type	Mo *K*α
μ (mm^−1^)	0.13
Crystal size (mm)	0.39 × 0.35 × 0.27

Data collection	
Diffractometer	Siemens/Bruker APEXII
Absorption correction	Multi-scan (*SADABS*; Bruker, 2008 ▸)
*T*_min_, *T*_max_	0.718, 0.746
No. of measured, independent and observed [*I* > 2σ(*I*)] reflections	29851, 5708, 4350
*R*_int_	0.047
(sin θ/λ)_max_ (Å^−1^)	0.639

Refinement	
*R*[*F* ^2^ > 2σ(*F* ^2^)], *wR*(*F* ^2^), *S*	0.046, 0.123, 1.02
No. of reflections	5708
No. of parameters	291
H-atom treatment	H-atom parameters constrained
Δρ_max_, Δρ_min_ (e Å^−3^)	0.36, −0.39

Computer programs: *APEX2* and *SAINT* (Bruker, 2008 ▸), *SHELXT2014* (Sheldrick, 2015*a* ▸), *SHELXL2016* (Sheldrick, 2015*b* ▸), *ORTEP-3 for Windows* (Farrugia, 2012 ▸), *PLATON* (Spek, 2009 ▸) and *publCIF* (Westrip, 2010 ▸).

## supplementary crystallographic information

### Crystal data

**Table:**

C_26_H_38_N_5_P	*F*(000) = 976
*M**_r_* = 451.58	*D*_x_ = 1.149 Mg m^−^^3^
Monoclinic, *P*2_1_/*n*	Mo *K*α radiation, λ = 0.71073 Å
*a* = 10.0148 (12) Å	Cell parameters from 5265 reflections
*b* = 17.343 (2) Å	θ = 2.2–25.2°
*c* = 15.6270 (19) Å	µ = 0.13 mm^−^^1^
β = 105.948 (2)°	*T* = 150 K
*V* = 2609.7 (5) Å^3^	Block, colourless
*Z* = 4	0.39 × 0.35 × 0.27 mm

### Data collection

**Table:**

Siemens/Bruker APEXII diffractometer	4350 reflections with *I* > 2σ(*I*)
Detector resolution: 66 pixels mm^-1^	*R*_int_ = 0.047
φ and ω scans	θ_max_ = 27.0°, θ_min_ = 1.8°
Absorption correction: multi-scan (SADABS; Bruker, 2008)	*h* = −12→12
*T*_min_ = 0.718, *T*_max_ = 0.746	*k* = −22→22
29851 measured reflections	*l* = −19→19
5708 independent reflections	

### Refinement

**Table:**

Refinement on *F*^2^	Primary atom site location: structure-invariant direct methods
Least-squares matrix: full	Hydrogen site location: inferred from neighbouring sites
*R*[*F*^2^ > 2σ(*F*^2^)] = 0.046	H-atom parameters constrained
*wR*(*F*^2^) = 0.123	*w* = 1/[σ^2^(*F*_o_^2^) + (0.0515*P*)^2^ + 1.4293*P*] where *P* = (*F*_o_^2^ + 2*F*_c_^2^)/3
*S* = 1.02	(Δ/σ)_max_ = 0.001
5708 reflections	Δρ_max_ = 0.36 e Å^−^^3^
291 parameters	Δρ_min_ = −0.39 e Å^−^^3^
0 restraints	

### Special details

**Table:**

Geometry. All esds (except the esd in the dihedral angle between two l.s. planes) are estimated using the full covariance matrix. The cell esds are taken into account individually in the estimation of esds in distances, angles and torsion angles; correlations between esds in cell parameters are only used when they are defined by crystal symmetry. An approximate (isotropic) treatment of cell esds is used for estimating esds involving l.s. planes.

### Fractional atomic coordinates and isotropic or equivalent isotropic displacement parameters (Å^2^)

**Table:**

	*x*	*y*	*z*	*U*_iso_*/*U*_eq_	
P1	0.59945 (4)	0.54203 (3)	0.26470 (3)	0.02022 (12)	
N1	0.79052 (16)	0.53002 (10)	0.29154 (11)	0.0320 (3)	
N2	0.84392 (16)	0.55461 (10)	0.23965 (11)	0.0320 (3)	
N3	0.9030 (2)	0.57761 (13)	0.19098 (13)	0.0507 (5)	
N4	0.54371 (15)	0.45109 (8)	0.24970 (9)	0.0219 (3)	
N5	0.57675 (15)	0.54345 (8)	0.36653 (9)	0.0213 (3)	
C1	0.5191 (3)	0.41282 (12)	0.32687 (13)	0.0425 (6)	
H1A	0.574471	0.364849	0.340333	0.051*	
H1B	0.419615	0.399379	0.315259	0.051*	
C2	0.5609 (2)	0.46709 (11)	0.40234 (13)	0.0364 (5)	
H2A	0.489221	0.468627	0.435128	0.044*	
H2B	0.649678	0.450354	0.443932	0.044*	
C3	0.51337 (18)	0.41222 (10)	0.16538 (11)	0.0231 (4)	
C4	0.60771 (19)	0.35813 (11)	0.14988 (12)	0.0274 (4)	
C5	0.5759 (2)	0.32088 (13)	0.06777 (14)	0.0413 (5)	
H5	0.638572	0.283914	0.055981	0.050*	
C6	0.4543 (2)	0.33718 (15)	0.00350 (14)	0.0492 (6)	
H6	0.434281	0.311734	−0.052431	0.059*	
C7	0.3621 (2)	0.38992 (13)	0.01970 (13)	0.0420 (5)	
H7	0.278444	0.400226	−0.025191	0.050*	
C8	0.38869 (19)	0.42858 (11)	0.10071 (12)	0.0286 (4)	
C9	0.7420 (2)	0.33751 (12)	0.21946 (13)	0.0336 (5)	
H9	0.750243	0.371828	0.272092	0.040*	
C10	0.7379 (3)	0.25436 (14)	0.25043 (17)	0.0561 (7)	
H10A	0.731285	0.219361	0.200199	0.084*	
H10B	0.656980	0.247168	0.273363	0.084*	
H10C	0.822820	0.243131	0.297620	0.084*	
C11	0.8689 (2)	0.35145 (16)	0.18521 (17)	0.0519 (6)	
H11A	0.865868	0.316548	0.135398	0.078*	
H11B	0.953597	0.341772	0.233196	0.078*	
H11C	0.868644	0.404983	0.165078	0.078*	
C12	0.2812 (2)	0.48372 (12)	0.11812 (14)	0.0367 (5)	
H12	0.326243	0.513951	0.172950	0.044*	
C13	0.1612 (3)	0.43896 (19)	0.1354 (3)	0.1000 (14)	
H13A	0.093897	0.475020	0.148196	0.150*	
H13B	0.196137	0.404586	0.186406	0.150*	
H13C	0.116090	0.408329	0.082660	0.150*	
C14	0.2272 (4)	0.53980 (17)	0.0432 (2)	0.0820 (10)	
H14A	0.177132	0.511669	−0.010362	0.123*	
H14B	0.305181	0.567885	0.031406	0.123*	
H14C	0.164104	0.576421	0.059862	0.123*	
C15	0.58170 (17)	0.61147 (10)	0.42027 (11)	0.0207 (3)	
C16	0.46359 (18)	0.65928 (10)	0.40491 (11)	0.0219 (4)	
C17	0.46834 (19)	0.72263 (10)	0.46045 (12)	0.0274 (4)	
H17	0.389882	0.755641	0.450633	0.033*	
C18	0.5844 (2)	0.73839 (11)	0.52929 (12)	0.0305 (4)	
H18	0.585062	0.781626	0.566744	0.037*	
C19	0.6994 (2)	0.69150 (10)	0.54386 (12)	0.0281 (4)	
H19	0.779039	0.703016	0.591430	0.034*	
C20	0.70149 (18)	0.62737 (10)	0.49017 (11)	0.0231 (4)	
C21	0.33327 (18)	0.64485 (11)	0.32984 (12)	0.0277 (4)	
H21	0.331695	0.589036	0.313540	0.033*	
C22	0.2000 (2)	0.66235 (13)	0.35634 (17)	0.0453 (6)	
H22A	0.191522	0.718159	0.363168	0.068*	
H22B	0.203732	0.636691	0.412819	0.068*	
H22C	0.119651	0.643396	0.309992	0.068*	
C23	0.3355 (2)	0.69207 (13)	0.24749 (14)	0.0412 (5)	
H23A	0.254521	0.678656	0.198252	0.062*	
H23B	0.420602	0.680639	0.230557	0.062*	
H23C	0.332647	0.747141	0.260929	0.062*	
C24	0.83013 (19)	0.57663 (11)	0.51126 (12)	0.0285 (4)	
H24	0.815047	0.535143	0.465149	0.034*	
C25	0.8521 (2)	0.53809 (13)	0.60211 (14)	0.0442 (5)	
H25A	0.769090	0.508486	0.602823	0.066*	
H25B	0.869085	0.577684	0.648599	0.066*	
H25C	0.932307	0.503398	0.613113	0.066*	
C26	0.9599 (2)	0.62141 (13)	0.50874 (16)	0.0432 (5)	
H26A	0.946650	0.644086	0.449550	0.065*	
H26B	1.039822	0.586419	0.521593	0.065*	
H26C	0.976794	0.662518	0.553468	0.065*	

### Atomic displacement parameters (Å^2^)

**Table:**

	*U*^11^	*U*^22^	*U*^33^	*U*^12^	*U*^13^	*U*^23^
P1	0.0210 (2)	0.0213 (2)	0.0179 (2)	−0.00023 (17)	0.00470 (16)	0.00068 (17)
N1	0.0236 (6)	0.0394 (7)	0.0296 (6)	0.0011 (5)	0.0017 (4)	−0.0038 (5)
N2	0.0236 (6)	0.0394 (7)	0.0296 (6)	0.0011 (5)	0.0017 (4)	−0.0038 (5)
N3	0.0390 (11)	0.0744 (15)	0.0439 (11)	−0.0083 (10)	0.0201 (9)	−0.0003 (10)
N4	0.0268 (8)	0.0214 (7)	0.0180 (7)	−0.0014 (6)	0.0070 (6)	−0.0002 (6)
N5	0.0271 (8)	0.0186 (7)	0.0191 (7)	0.0007 (6)	0.0078 (6)	0.0005 (6)
C1	0.0801 (17)	0.0260 (10)	0.0300 (11)	−0.0088 (10)	0.0293 (11)	−0.0024 (8)
C2	0.0631 (14)	0.0236 (10)	0.0229 (9)	−0.0099 (9)	0.0125 (9)	0.0016 (8)
C3	0.0262 (9)	0.0233 (9)	0.0200 (8)	−0.0019 (7)	0.0067 (7)	−0.0023 (7)
C4	0.0286 (10)	0.0290 (10)	0.0239 (9)	0.0018 (8)	0.0060 (7)	−0.0042 (7)
C5	0.0395 (12)	0.0490 (13)	0.0339 (11)	0.0079 (10)	0.0077 (9)	−0.0162 (10)
C6	0.0501 (14)	0.0641 (16)	0.0275 (11)	0.0046 (12)	0.0009 (10)	−0.0212 (11)
C7	0.0367 (12)	0.0535 (14)	0.0277 (10)	0.0043 (10)	−0.0050 (9)	−0.0066 (9)
C8	0.0265 (9)	0.0280 (10)	0.0285 (10)	0.0009 (7)	0.0029 (8)	−0.0011 (8)
C9	0.0325 (11)	0.0347 (11)	0.0301 (10)	0.0094 (8)	0.0027 (8)	−0.0077 (8)
C10	0.0661 (17)	0.0387 (13)	0.0538 (15)	0.0155 (12)	−0.0002 (13)	0.0041 (11)
C11	0.0322 (12)	0.0669 (17)	0.0542 (15)	0.0099 (11)	0.0081 (10)	−0.0126 (12)
C12	0.0262 (10)	0.0320 (11)	0.0448 (12)	0.0055 (8)	−0.0022 (9)	−0.0070 (9)
C13	0.073 (2)	0.065 (2)	0.192 (4)	0.0142 (17)	0.086 (3)	0.009 (2)
C14	0.098 (3)	0.0568 (19)	0.082 (2)	0.0371 (17)	0.0091 (19)	0.0182 (16)
C15	0.0260 (9)	0.0196 (8)	0.0177 (8)	−0.0012 (7)	0.0080 (7)	0.0004 (6)
C16	0.0245 (9)	0.0198 (8)	0.0223 (9)	−0.0013 (7)	0.0079 (7)	0.0033 (7)
C17	0.0320 (10)	0.0206 (9)	0.0304 (10)	0.0035 (7)	0.0100 (8)	0.0000 (7)
C18	0.0400 (11)	0.0231 (9)	0.0285 (10)	−0.0017 (8)	0.0093 (8)	−0.0069 (8)
C19	0.0321 (10)	0.0280 (10)	0.0219 (9)	−0.0041 (8)	0.0035 (7)	−0.0014 (7)
C20	0.0261 (9)	0.0222 (9)	0.0212 (8)	−0.0006 (7)	0.0068 (7)	0.0025 (7)
C21	0.0247 (9)	0.0230 (9)	0.0329 (10)	−0.0002 (7)	0.0036 (8)	−0.0010 (8)
C22	0.0250 (11)	0.0445 (13)	0.0645 (15)	−0.0016 (9)	0.0090 (10)	−0.0103 (11)
C23	0.0414 (12)	0.0395 (12)	0.0344 (11)	0.0035 (9)	−0.0038 (9)	0.0086 (9)
C24	0.0284 (10)	0.0263 (9)	0.0272 (10)	0.0026 (8)	0.0015 (7)	−0.0014 (8)
C25	0.0451 (13)	0.0438 (13)	0.0366 (12)	0.0078 (10)	−0.0009 (10)	0.0118 (10)
C26	0.0291 (11)	0.0429 (13)	0.0576 (14)	0.0040 (9)	0.0120 (10)	0.0013 (11)

### Geometric parameters (Å, º)

**Table:**

P1—N4	1.6680 (15)	C12—H12	1.0000
P1—N5	1.6684 (14)	C13—H13A	0.9800
P1—N1	1.8547 (16)	C13—H13B	0.9800
N1—N2	1.168 (2)	C13—H13C	0.9800
N2—N3	1.155 (2)	C14—H14A	0.9800
N4—C3	1.436 (2)	C14—H14B	0.9800
N4—C1	1.456 (2)	C14—H14C	0.9800
N5—C15	1.441 (2)	C15—C16	1.410 (2)
N5—C2	1.463 (2)	C15—C20	1.410 (2)
C1—C2	1.477 (3)	C16—C17	1.393 (2)
C1—H1A	0.9900	C16—C21	1.517 (2)
C1—H1B	0.9900	C17—C18	1.377 (3)
C2—H2A	0.9900	C17—H17	0.9500
C2—H2B	0.9900	C18—C19	1.377 (3)
C3—C4	1.399 (2)	C18—H18	0.9500
C3—C8	1.403 (2)	C19—C20	1.397 (2)
C4—C5	1.393 (3)	C19—H19	0.9500
C4—C9	1.521 (3)	C20—C24	1.520 (2)
C5—C6	1.378 (3)	C21—C23	1.531 (3)
C5—H5	0.9500	C21—C22	1.532 (3)
C6—C7	1.372 (3)	C21—H21	1.0000
C6—H6	0.9500	C22—H22A	0.9800
C7—C8	1.392 (3)	C22—H22B	0.9800
C7—H7	0.9500	C22—H22C	0.9800
C8—C12	1.519 (3)	C23—H23A	0.9800
C9—C10	1.525 (3)	C23—H23B	0.9800
C9—C11	1.528 (3)	C23—H23C	0.9800
C9—H9	1.0000	C24—C26	1.524 (3)
C10—H10A	0.9800	C24—C25	1.529 (3)
C10—H10B	0.9800	C24—H24	1.0000
C10—H10C	0.9800	C25—H25A	0.9800
C11—H11A	0.9800	C25—H25B	0.9800
C11—H11B	0.9800	C25—H25C	0.9800
C11—H11C	0.9800	C26—H26A	0.9800
C12—C14	1.503 (3)	C26—H26B	0.9800
C12—C13	1.516 (4)	C26—H26C	0.9800
			
N4—P1—N5	91.14 (7)	C12—C13—H13B	109.5
N4—P1—N1	102.14 (8)	H13A—C13—H13B	109.5
N5—P1—N1	100.86 (7)	C12—C13—H13C	109.5
N2—N1—P1	116.29 (14)	H13A—C13—H13C	109.5
N3—N2—N1	176.6 (2)	H13B—C13—H13C	109.5
C3—N4—C1	120.31 (14)	C12—C14—H14A	109.5
C3—N4—P1	123.43 (11)	C12—C14—H14B	109.5
C1—N4—P1	116.17 (12)	H14A—C14—H14B	109.5
C15—N5—C2	120.53 (14)	C12—C14—H14C	109.5
C15—N5—P1	125.17 (11)	H14A—C14—H14C	109.5
C2—N5—P1	114.15 (12)	H14B—C14—H14C	109.5
N4—C1—C2	107.20 (16)	C16—C15—C20	120.81 (15)
N4—C1—H1A	110.3	C16—C15—N5	119.29 (15)
C2—C1—H1A	110.3	C20—C15—N5	119.82 (15)
N4—C1—H1B	110.3	C17—C16—C15	118.32 (16)
C2—C1—H1B	110.3	C17—C16—C21	119.13 (16)
H1A—C1—H1B	108.5	C15—C16—C21	122.54 (15)
N5—C2—C1	107.95 (15)	C18—C17—C16	121.37 (17)
N5—C2—H2A	110.1	C18—C17—H17	119.3
C1—C2—H2A	110.1	C16—C17—H17	119.3
N5—C2—H2B	110.1	C17—C18—C19	120.01 (17)
C1—C2—H2B	110.1	C17—C18—H18	120.0
H2A—C2—H2B	108.4	C19—C18—H18	120.0
C4—C3—C8	121.38 (16)	C18—C19—C20	121.38 (17)
C4—C3—N4	119.40 (15)	C18—C19—H19	119.3
C8—C3—N4	119.21 (15)	C20—C19—H19	119.3
C5—C4—C3	118.43 (17)	C19—C20—C15	118.11 (16)
C5—C4—C9	118.87 (17)	C19—C20—C24	118.84 (16)
C3—C4—C9	122.69 (16)	C15—C20—C24	123.02 (15)
C6—C5—C4	120.59 (19)	C16—C21—C23	110.73 (15)
C6—C5—H5	119.7	C16—C21—C22	112.73 (16)
C4—C5—H5	119.7	C23—C21—C22	109.64 (17)
C7—C6—C5	120.47 (19)	C16—C21—H21	107.9
C7—C6—H6	119.8	C23—C21—H21	107.9
C5—C6—H6	119.8	C22—C21—H21	107.9
C6—C7—C8	121.22 (19)	C21—C22—H22A	109.5
C6—C7—H7	119.4	C21—C22—H22B	109.5
C8—C7—H7	119.4	H22A—C22—H22B	109.5
C7—C8—C3	117.90 (17)	C21—C22—H22C	109.5
C7—C8—C12	119.86 (17)	H22A—C22—H22C	109.5
C3—C8—C12	122.18 (17)	H22B—C22—H22C	109.5
C4—C9—C10	110.70 (18)	C21—C23—H23A	109.5
C4—C9—C11	111.68 (17)	C21—C23—H23B	109.5
C10—C9—C11	110.99 (19)	H23A—C23—H23B	109.5
C4—C9—H9	107.8	C21—C23—H23C	109.5
C10—C9—H9	107.8	H23A—C23—H23C	109.5
C11—C9—H9	107.8	H23B—C23—H23C	109.5
C9—C10—H10A	109.5	C20—C24—C26	112.19 (16)
C9—C10—H10B	109.5	C20—C24—C25	110.59 (16)
H10A—C10—H10B	109.5	C26—C24—C25	109.90 (17)
C9—C10—H10C	109.5	C20—C24—H24	108.0
H10A—C10—H10C	109.5	C26—C24—H24	108.0
H10B—C10—H10C	109.5	C25—C24—H24	108.0
C9—C11—H11A	109.5	C24—C25—H25A	109.5
C9—C11—H11B	109.5	C24—C25—H25B	109.5
H11A—C11—H11B	109.5	H25A—C25—H25B	109.5
C9—C11—H11C	109.5	C24—C25—H25C	109.5
H11A—C11—H11C	109.5	H25A—C25—H25C	109.5
H11B—C11—H11C	109.5	H25B—C25—H25C	109.5
C14—C12—C13	109.7 (2)	C24—C26—H26A	109.5
C14—C12—C8	112.9 (2)	C24—C26—H26B	109.5
C13—C12—C8	110.17 (19)	H26A—C26—H26B	109.5
C14—C12—H12	108.0	C24—C26—H26C	109.5
C13—C12—H12	108.0	H26A—C26—H26C	109.5
C8—C12—H12	108.0	H26B—C26—H26C	109.5
C12—C13—H13A	109.5		
			
N4—P1—N1—N2	113.83 (16)	C3—C4—C9—C10	113.2 (2)
N5—P1—N1—N2	−152.62 (15)	C5—C4—C9—C11	58.4 (3)
N5—P1—N4—C3	169.76 (14)	C3—C4—C9—C11	−122.6 (2)
N1—P1—N4—C3	−88.87 (14)	C7—C8—C12—C14	−48.6 (3)
N5—P1—N4—C1	−6.93 (16)	C3—C8—C12—C14	134.2 (2)
N1—P1—N4—C1	94.44 (16)	C7—C8—C12—C13	74.3 (3)
N4—P1—N5—C15	−168.59 (14)	C3—C8—C12—C13	−102.9 (3)
N1—P1—N5—C15	88.82 (14)	C2—N5—C15—C16	−103.4 (2)
N4—P1—N5—C2	15.88 (14)	P1—N5—C15—C16	81.30 (19)
N1—P1—N5—C2	−86.71 (14)	C2—N5—C15—C20	73.4 (2)
C3—N4—C1—C2	179.70 (17)	P1—N5—C15—C20	−101.83 (17)
P1—N4—C1—C2	−3.5 (2)	C20—C15—C16—C17	0.1 (2)
C15—N5—C2—C1	163.95 (17)	N5—C15—C16—C17	176.90 (15)
P1—N5—C2—C1	−20.3 (2)	C20—C15—C16—C21	179.24 (16)
N4—C1—C2—N5	14.1 (3)	N5—C15—C16—C21	−3.9 (2)
C1—N4—C3—C4	−79.8 (2)	C15—C16—C17—C18	−0.5 (3)
P1—N4—C3—C4	103.69 (18)	C21—C16—C17—C18	−179.74 (17)
C1—N4—C3—C8	99.4 (2)	C16—C17—C18—C19	0.6 (3)
P1—N4—C3—C8	−77.2 (2)	C17—C18—C19—C20	−0.2 (3)
C8—C3—C4—C5	0.6 (3)	C18—C19—C20—C15	−0.2 (3)
N4—C3—C4—C5	179.73 (18)	C18—C19—C20—C24	−178.33 (17)
C8—C3—C4—C9	−178.35 (18)	C16—C15—C20—C19	0.3 (2)
N4—C3—C4—C9	0.8 (3)	N5—C15—C20—C19	−176.51 (15)
C3—C4—C5—C6	0.1 (3)	C16—C15—C20—C24	178.31 (16)
C9—C4—C5—C6	179.1 (2)	N5—C15—C20—C24	1.5 (2)
C4—C5—C6—C7	−0.6 (4)	C17—C16—C21—C23	84.6 (2)
C5—C6—C7—C8	0.4 (4)	C15—C16—C21—C23	−94.5 (2)
C6—C7—C8—C3	0.3 (3)	C17—C16—C21—C22	−38.6 (2)
C6—C7—C8—C12	−177.1 (2)	C15—C16—C21—C22	142.23 (18)
C4—C3—C8—C7	−0.8 (3)	C19—C20—C24—C26	−58.9 (2)
N4—C3—C8—C7	−179.90 (18)	C15—C20—C24—C26	123.11 (19)
C4—C3—C8—C12	176.48 (18)	C19—C20—C24—C25	64.2 (2)
N4—C3—C8—C12	−2.6 (3)	C15—C20—C24—C25	−113.80 (19)
C5—C4—C9—C10	−65.8 (3)		

### Hydrogen-bond geometry (Å, º)

**Table:**

*D*—H···*A*	*D*—H	H···*A*	*D*···*A*	*D*—H···*A*
C9—H9···N4	1.00	2.43	2.926 (2)	110
C12—H12···N4	1.00	2.44	2.913 (2)	109
C21—H21···N5	1.00	2.49	2.932 (2)	106
C24—H24···N1	1.00	2.66	3.443 (3)	136
C24—H24···N5	1.00	2.46	2.955 (2)	110
C22—H22*C*···N3^i^	0.98	2.69	3.669 (3)	174

Symmetry code: (i) *x*−1, *y*, *z*.
